# FTO gene SNPs associated with extreme obesity in cases, controls and extremely discordant sister pairs

**DOI:** 10.1186/1471-2350-9-4

**Published:** 2008-01-24

**Authors:** R Arlen Price, Wei-Dong Li, Hongyu Zhao

**Affiliations:** 1 Center for Neurobiology and Behavior, Department of Psychiatry, University of Pennsylvania, Philadelphia, Pennsylvania, USA; 2 Department of Epidemiology and Public Health, Yale University School of Medicine, New Haven, Connecticut, USA

## Abstract

**Background:**

FTO is a gene located in chromosome region 16q12.2. Recently two studies have found associations of several single nucleotide polymorphisms (SNPs) in FTO with body mass index (BMI) and obesity, particularly rs1421085, rs17817449, and rs9939609.

**Methods:**

We examined these three SNPs in 583 extremely obese women with current BMI greater than 35 kg/m^2 ^and lifetime BMI greater than 40 kg/m^2^, and 544 controls who were currently normal weight (BMI<25 kg/m^2^) and had never been overweight during their lifetimes.

**Results:**

We detected highly significant associations of obesity with alleles in all three SNPs (p < 10^-9^). The strongest association was with rs1421085 (p = 3.04 × 10^-10^, OR = 1.75, CI = 1.47–2.08). A subset of 99 cases had extremely discordant sisters with BMI<25 kg/m^2^. The discordant sisters differed in allele and genotype frequencies in parallel with the overall case and control sample. The strongest association was with rs17817449 (z = 3.57, p = 3.6 × 10^-4^).

**Conclusion:**

These results suggest common variability in FTO is associated with increased obesity risk or resistance and may in part account for differences between closely related individuals.

## Background

Obesity has become the most common human disorder and it poses a serious threat to health and quality of life through its association with diabetes, hypertension, cardiovascular disease, and cancer. Rates have been increasing during the past century, particularly during the past two decades, and show no sign of leveling off. The average body mass index (BMI, weight in kg/height in m^2^) of the U.S. population is well into the overweight range (BMI = 28.1 kg/m^2^), more than 30% of the people in the United States are obese (BMI ≥ 30 kg/m^2^) and more than 64% are overweight (BMI > 25 kg/m^2^)[1]. Recent predictions are for a reduction in life expectancy and quality of life for the next generation [2].

Recently, a genome wide association study revealed an association with a common variant in single nucleotide polymorphisms located in intron 1 of FTO, a gene with previously unknown function located in chromosome region 16q12.2 [3]. A second study found associations with nearby SNPs[4]. The 3 primary SNPs, rs9939609 in the Frayling et al. [3] study and rs1421085 and rs17817449 in the Dina et al study[4], are located within 20 kb and are in strong linkage disequilibrium (LD). The FTO association is notable because of its strength and the consistency of replication across several samples, both within and between studies. For example, FTO associations with physical activity related body fat accumulation [5] and obesity related traits [6,7] were found in studies having large sample sizes. To date, the obesity association has been found only in Europeans or individuals of European descent. FTO was not associated with obesity in Han Chinese [8] or with six Oceanic populations [9]. Field et al. [10] tested FTO gene variants in type 1 diabetes cases and controls, however, no significant association was found. The association with type II diabetes is mediated entirely by BMI [3].

In this report we examined three SNPs to determine whether FTO variability could distinguish between extremely obese cases and thin controls. In addition, we extended our investigation to examine extremely discordant sister pairs, because the shared genetic background may be useful in future attempts to identify functional variants.

## Methods

### Cases and controls

Cases were obese women (BMI ≥ 35 kg/m^2^) with a lifetime BMI >40 kg/m^2^. Independent controls were selected who had a current and lifetime BMI ≤ 25. All cases and controls were non-Hispanic Caucasians. Sample characteristics are summarized in Table 1. The individuals in the samples were of approximately the same age but differed in average BMI by 29 kg/m^2^. As would be expected for extreme obesity, most cases had an early onset. The median age at obesity onset was 12 and 90% had an onset prior to age 26. This result is consistent with the observation of Frayling et al. [3] of an FTO association in children as young as seven years.

**Table 1:** Clinical characteristics of cases/controls and discordant sister pairs

	N	Minimum	Maximum	Mean	Std. Deviation
**Controls**					
Age	544	16	65	42.63	8.75
BMI	544	15.99	24.93	20.77	1.79
% Fat	534	7.50	40.00	23.65	5.61
**Cases**					
Age	583	18	64	41.05	9.42
BMI	583	35.08	96.95	49.41	8.81
% Fat	529	30.70	70.70	50.01	5.92
Onset Age	462	0.1	55	13.90	9.01
**Thin Sisters**					
Age	99	14	56	37.61	9.10
BMI	99	17.01	24.90	21.80	1.88
% Fat	81	15.10	38.40	26.41	5.58
**Obese Sisters**					
Age	99	17	65	39.55	9.27
BMI	99	35.08	71.35	47.46	8.10
% Fat	89	37.00	58.90	49.36	5.18
Onset Age	78	1	42	14.04	8.10
**Differences (Obese-Thin)**					
Age	99	-19.00	18.00	1.94	6.39
BMI	99	13.14	50.26	25.65	8.27
% Fat	79	6.30	40.20	23.33	7.41

All subjects in the three samples gave informed consent, and the protocol was approved by the Committee on Studies Involving Human Beings at the University of Pennsylvania.

### Discordant Sister Pairs

A subset of the case sample had extremely discordant sisters. Characteristics of the sister pairs are presented in Table 1. All thin sisters had BMI < 25 kg/m^2 ^and had never been obese (BMI ≥ 27 kg/m^2^), while obese sisters had BMI ≥ 35 kg/m^2^. The sisters were similar in age but differed markedly in BMI by an average of 26 kg/m^2^. This difference is more than double the average BMI of the thin sisters, making the comparison comparable in magnitude to some animal models (Table 1). The percentage body fat of normal weight and obese sisters were 26.41 ± 5.58 and 49.36 ± 5.18, respectively.

### SNP Markers

The three SNPs, rs1421085, rs17817449, and rs9939609, are located within 19.6 kb in intron 1 of the FTO gene and are in strong LD (pairwise r^2^>0.97) based on our samples. There are two primary haplotypes, C-G-A (42.0 %) and T-T-T (55.5 %). The T-C-T haplotype was observed at 1.7 % and 5 others had very low frequencies. Pairwise linkage disequilibrium measures used Proc Allele and haplotype analyses the Proc Haplotype EM algorithm [11] in SAS 9.1.

### Genotyping

DNA was extracted from whole blood or lymphoblastoid cell lines using a high salt method. PCR reactions and genotyping were completed using an Applied Biosystems (ABI, Foster City, CA) TaqMan 7300 system. SNP primers and probes were provided by ABI Assays-on-Demand. Detailed conditions are available on request.

### Statistical analyses

Descriptive statistics, allele and genotype frequencies were calculated using SPSS 15.0. Tests of Hardy-Weinberg (HW) equilibrium were based on a Chi Square goodness-of-fit test programmed in Excel. The program Structure was used to test for possible population structure. For this purpose, more than 300 SNP markers were available for cases, while a subset of 37 were available for both cases and controls. Association analyses were completed using the Case Control procedure in SAS 9.1, with the overall association with genotype based on the Armitage trend test and odds ratios based on allele counts, reflecting additive effects.

For discordant sister pairs, mean values for BMI and percent fat were compared using one-way ANOVA and analysis of covariance used General Linear Models in SPSS 15.0. Association analyses for discordant sisters used the sibling transmission disequilibrium test (S-TDT) [12]

### Power

For the case/control comparisons, power was estimated using parameters from the original Frayling et al. [3] study, since that study had the largest sample size. Values reported by Dina et al. [4] were similar. Assuming a risk allele frequency of .45, obesity prevalence = 0.2 (BMI > 35 kg/m^2^), linkage disequilibrium (D') = 0.80, relative risk (RR) = 1.67, p < .05, and additive, dominant or recessive expression, we have greater than 80% power with 520 unpaired cases and controls, the minimum number of individuals with genotypes. This estimate considers only the risk for the upper extreme phenotype, and conservatively assumes a population prevalence based on current BMI>35 rather than lifetime BMI>40. Power is higher still if risk and protection are considered in the respective tails of the distribution (analyses not shown).

## Results

### Genotyping

Genotype frequencies for controls, cases and the combined sample were all in Hardy-Weinberg equilibrium, and there was no evidence of structure (analyses not shown). Genotype and allele frequencies for cases and controls are given in the Table 2. Obese cases have consistently higher minor allele frequencies that are near or above 0.5, while those for controls were about 0.13 lower. The genotype frequencies were similar for controls and thin sisters and minor allele frequencies were 9–12% lower than their obese sibling. Significance for case-control and thin-obese sister allele frequency comparisons are given in the right column. All reach statistical significance except for rs1421085 in discordant sisters.

**Table 2:** Genotype and allele frequencies of three FTO SNPs in cases/controls and discordant sisters. Significance for case-control and thin-obese sister allele frequency comparisons are given in the right column. Genotype comparisons are presented in Table 3.

	N	Genotypes			Alleles		Allele Freq. Difference
**rs1421085**		TT	TC	CC	T	C	
Controls	521	.370	.518	.111	.630	.370	
Cases	523	.256	.474	.270	.493	.507	p = 3.5 × 10^-10^
Thin Sisters	92	.337	.554	.109	.614	.386	
Obese Sisters	93	.247	.548	.204	.522	.478	p = 0.07
**rs17817449**		TT	TG	GG	T	G	
Controls	522	.393	.496	.111	.641	.359	
Cases	527	.275	.469	.256	.509	.491	p = 1.2 × 10^-9^
Thin Sisters	98	.347	.571	.082	.633	.367	
Obese Sisters	93	.258	.516	.226	.516	.484	p = 0.02
**rs9939609**		TT	TA	AA	T	A	
Controls	520	.398	.494	.108	.645	.355	
Cases	527	.288	.459	.252	.518	.482	p = 3.7 × 10^-9^
Thin Sisters	98	.367	.541	.092	.638	.362	
Obese Sisters	92	.272	.489	.239	.516	.484	p = 0.02

### Association study in cases and controls

The results from the association analyses for cases and controls are presented in Table 3. Results were highly significant (p < 10^-9^) for all three SNPs. The strongest association was with rs1421985 (p = 3.04 × 10^-10^, OR = 1.75, CI = 1.47–2.08) (Table 3). Considering all 8 observed haplotypes the overall χ^2 ^for association with case status was 50.69 (df = 7, p = 1.07 × 10^-8^). For the two common haplotypes C-G-A and T-T-T the values were 36.47 (df = 7, p = 1.55 × 10^-9^) and 36.50 (df = 7, p = 1.53 × 10^-9^), respectively.

**Table 3:** Association studies of three FTO SNPs in cases and controls

SNP	N Controls	N Cases	Armitage χ^2^	p	Additive OR (CI)
**Cases and controls**					
rs1421085	521	523	39.65	3.04 × 10^-10^	1.75 (1.47–2.08)
rs17817449	522	527	36.55	1.51 × 10^-9^	1.72 (1.44–2.05)
rs9939609	520	527	34.06	5.34 × 10^-9^	1.69 (1.42–2.02)

### Association studies in extremely discordant sister pairs

Results from association analyses for discordant sister pairs are presented in Table 4. All associations are significant and parallel those for allele frequencies (Table 2) and associations in cases and controls (Table 3).

**Table 4:** Association analyses of extremely discordant sibling pairs.

Obese	Thin			Z	P-value
rs1421085	TT	TC	CC		
TT	15	6	0	2.14	p = 0.032
TC	14	32	2		
CC	2	7	8		
rs17817449	TT	TG	GG		
TT	18	6	0	3.57	p = 3.6 × 10^-4^
TG	13	34	1		
GG	2	11	8		
rs9939609	TT	TA	AA		
TT	18	7	0	3.09	p = 0.002
TA	14	30	1		
AA	2	12	8		

## Discussion

The results of this study strongly replicate and extend those from previous reports of an association between FTO and human obesity. The effect size was large, with odds ratios to 1.69–1.75 in cases and controls and 1.46–1.65 in discordant sisters. These values are similar to those reported previously [3,4]. The consistency and strength of the associations along with the high frequencies of the risk alleles/haplotype among extremely obese individuals suggest the effects of this gene, while still unknown, are common and pervasive. Given the history of non-replications for other gene associations, the consistency and strength of the replication is particularly notable. Moreover, because of the very large sample sizes in the original studies [3,4] one might reasonably have expected it would be difficult to find the association with moderate sample sizes like those of the current study. However, the effects are quite strong and detectible even in a relatively small sample of discordant sisters.

Extreme sampling from both tails of the phenotypic distribution is uncommon, because the screening process in sample selection requires much more effort. However, the increase in power to detect associations is substantial [13]. The design should select for both risk and protective variation in cases and controls, respectively The case-control sample of the current study is a powerful one and likely played a role in the strong replication, even though it is only of moderate size.

The use of extremely discordant sibling pairs is particularly uncommon because of the difficulty and expense in accruing such samples. However, pairs that differ in phenotype can increase power to identify associations, particularly when they also differ in genotype [14]. The results from this study indicate that FTO can have a substantial effect on obesity risk between individuals close in age, having similar rearing environments, and sharing half their genes identical by descent. While the sample sizes for discordant siblings may be too small for definitive conclusions, they are highly significant, consistent with other results, and can be particularly useful in isolating causative sequence variability once an overall association has been identified.

Although the role of FTO or nearby genes in susceptibility to obesity remains unknown, several recent studies have begun to shed light on its function. For example, FTO is highly expressed in hypothalamic nuclei that control eating behavior [15]. An intriguing observation is that it catalyzes Fe(II)- and 2OG- dependent DNA demethylation [15], although the role of FTO related DNA methylation in obesity is unknown. Further studies are needed to clarify the functional relationship of FTO with obesity susceptibility.

## Conclusion

These results suggest common variability in FTO is associated with increased obesity risk or resistance and may in part account for differences between closely related individuals.

## Abbreviations

BMI Body mass index

SNP Single nucleotide polymorphism

FTO Homolog of mouse *fatso *(fused toes) gene

LD Linkage disequilibrium

## Competing interests

The author(s) declare that they have no competing interests.

## Authors' contributions

RAP is PI of the projects that accrued the samples and performed the phenotyping. For this specific study, he conducted statistical analyses and drafted the manuscript. WDL participated in data collection, performed data analyses, conducted genotyping, and made contributions to the manuscript. HZ analyzed data, advised on statistical analyses, and contributed to the manuscript. All authors have read and approved the final manuscript.

## Pre-publication history

The pre-publication history for this paper can be accessed here:



## References

[B1] Hedley AA, Ogden CL, Johnson CL, Carroll MD, Curtin LR, Flegal KM (2004). Prevalence of overweight and obesity among US children, adolescents, and adults, 1999-2002. Jama.

[B2] Flegal KM, Graubard BI, Williamson DF, Gail MH (2005). Excess deaths associated with underweight, overweight, and obesity. Jama.

[B3] Frayling TM, Timpson NJ, Weedon MN, Zeggini E, Freathy RM, Lindgren CM, Perry JR, Elliott KS, Lango H, Rayner NW, Shields B, Harries LW, Barrett JC, Ellard S, Groves CJ, Knight B, Patch AM, Ness AR, Ebrahim S, Lawlor DA, Ring SM, Ben-Shlomo Y, Jarvelin MR, Sovio U, Bennett AJ, Melzer D, Ferrucci L, Loos RJ, Barroso I, Wareham NJ, Karpe F, Owen KR, Cardon LR, Walker M, Hitman GA, Palmer CN, Doney AS, Morris AD, Smith GD, Hattersley AT, McCarthy MI (2007). A common variant in the FTO gene is associated with body mass index and predisposes to childhood and adult obesity. Science.

[B4] Dina C, Meyre D, Gallina S, Durand E, Korner A, Jacobson P, Carlsson LM, Kiess W, Vatin V, Lecoeur C, Delplanque J, Vaillant E, Pattou F, Ruiz J, Weill J, Levy-Marchal C, Horber F, Potoczna N, Hercberg S, Le Stunff C, Bougneres P, Kovacs P, Marre M, Balkau B, Cauchi S, Chevre JC, Froguel P (2007). Variation in FTO contributes to childhood obesity and severe adult obesity. Nat Genet.

[B5] Andreasen CH, Stender-Petersen KL, Mogensen MS, Torekov SS, Wegner L, Andersen G, Nielsen AL, Albrechtsen A, Borch-Johnsen K, Rasmussen SS, Clausen JO, Sandbaek A, Lauritzen T, Hansen L, Jorgensen T, Pedersen O, Hansen T (2007). Low physical activity accentuates the effect of the FTO rs9939609 polymorphism on body fat accumulation. Diabetes.

[B6] Scuteri A, Sanna S, Chen WM, Uda M, Albai G, Strait J, Najjar S, Nagaraja R, Orru M, Usala G, Dei M, Lai S, Maschio A, Busonero F, Mulas A, Ehret GB, Fink AA, Weder AB, Cooper RS, Galan P, Chakravarti A, Schlessinger D, Cao A, Lakatta E, Abecasis GR (2007). Genome-Wide Association Scan Shows Genetic Variants in the FTO Gene Are Associated with Obesity-Related Traits. PLoS Genet.

[B7] Peeters A, Beckers S, Verrijken A, Roevens P, Peeters P, Van Gaal L, Van Hul W (2007). Variants in the FTO gene are associated with common obesity in the Belgian population. Mol Genet Metab.

[B8] Li H, Wu Y, Loos RJ, Hu FB, Liu Y, Wang J, Yu Z, Lin X (2007). Variants in FTO gene are not associated with obesity in a Chinese Han population. Diabetes.

[B9] Ohashi J, Naka I, Kimura R, Natsuhara K, Yamauchi T, Furusawa T, Nakazawa M, Ataka Y, Patarapotikul J, Nuchnoi P, Tokunaga K, Ishida T, Inaoka T, Matsumura Y, Ohtsuka R (2007). FTO polymorphisms in oceanic populations. J Hum Genet.

[B10] Field SF, Howson JM, Walker NM, Dunger DB, Todd JA (2007). Analysis of the obesity gene FTO in 14,803 type 1 diabetes cases and controls. Diabetologia.

[B11] Ott J (1977). Counting methods (EM algorithm) in human pedigree analysis: linkage and segregation analysis. Ann Hum Genet.

[B12] Spielman RS, Ewens WJ (1998). A sibship test for linkage in the presence of association: the sib transmission/disequilibrium test [see comments]. Am J Hum Genet.

[B13] Abecasis GR, Cookson WO, Cardon LR (2001). The power to detect linkage disequilibrium with quantitative traits in selected samples. Am J Hum Genet.

[B14] Wessel J, Schork AJ, Tiwari HK, Schork NJ (2007). Powerful designs for genetic association studies that consider twins and sibling pairs with discordant genotypes. Genet Epidemiol.

[B15] Gerken T, Girard CA, Tung YC, Webby CJ, Saudek V, Hewitson KS, Yeo GS, McDonough MA, Cunliffe S, McNeill LA, Galvanovskis J, Rorsman P, Robins P, Prieur X, Coll AP, Ma M, Jovanovic Z, Farooqi IS, Sedgwick B, Barroso I, Lindahl T, Ponting CP, Ashcroft FM, O'Rahilly S, Schofield CJ (2007). The obesity-associated FTO gene encodes a 2-oxoglutarate-dependent nucleic acid demethylase. Science.

